# Porphyrins for Probing Electrical Potential Across Lipid Bilayer Membranes by Second Harmonic Generation**

**DOI:** 10.1002/anie.201304515

**Published:** 2013-07-16

**Authors:** James E Reeve, Alex D Corbett, Igor Boczarow, Wojciech Kaluza, William Barford, Hagan Bayley, Tony Wilson, Harry L Anderson

**Affiliations:** Department of Chemistry, University of OxfordOxford OX1 3TA (UK); Department of Engineering Science, University of OxfordOxford OX1 3PJ (UK)

**Keywords:** dyes, membrane potential, membranes, nonlinear optics, porphyrinoids

## Abstract

Neurons communicate by using electrical signals, mediated by transient changes in the voltage across the plasma membrane. Optical techniques for visualizing these transmembrane potentials could revolutionize the field of neurobiology by allowing the spatial profile of electrical activity to be imaged in real time with high resolution, along individual neurons or groups of neurons within their native networks.^1^, ^2^ Second harmonic generation (SHG) is one of the most promising methods for imaging membrane potential, although so far this technique has only been demonstrated with a narrow range of dyes.^3^ Here we show that SHG from a porphyrin-based membrane probe gives a fast electro-optic response to an electric field which is about 5–10 times greater than that of conventional styryl dyes. Our results indicate that porphyrin dyes are promising probes for imaging membrane potential.

Studies of excitable cells, such as neurons and cardiac myocytes, require new methods for mapping changes in membrane potential, ideally with a voltage resolution of about 1 mV, a time resolution of 1 ms, and a spatial resolution of 1 μm.^1^, ^2^ Microelectrodes can be used to measure transmembrane potentials with excellent sensitivity and temporal resolution, but they do not provide subcellular spatial resolution. Fluorescent calcium indicators are widely used to probe membrane potential indirectly, through the intracellular Ca^2+^ concentration. However, changes in calcium concentration do not accurately reflect voltage transients.^4^ Fluorescent voltage-sensitive dyes were first developed 30 years ago,^5^ and recent advances in the molecular design of these dyes^6^–^10^ have made it possible to track the spatial evolution of action potentials.^11^–^14^ Compared with fluorescence, SHG imaging has several advantages as a technique for probing membrane potential:^3^, ^15^–^21^ SHG has a greater intrinsic sensitivity to electric fields than fluorescence,^19^–^21^ and it is only produced by noncentrosymmetric molecules in noncentrosymmetric environments, thus making it ideal for probing interfaces such as lipid bilayers. Similar to two-photon-excited fluorescence, SHG is a two-photon nonlinear optical effect, and it brings the advantages of depth penetration associated with multiphoton microscopy.^22^ The main disadvantage of SHG is that it often gives lower intensity than fluorescence; there is a need for the design of brighter, more voltage-sensitive SHG dyes.^3^, ^23^ Styryl dyes and retinal chromophores have been shown to exhibit voltage-sensitive SHG when localized in plasma membranes,^15^, ^24^ and the voltage sensitivity of their SHG is greater than that of their fluorescence.^19^–^21^ Recently we reported that amphiphilic porphyrins such as JR1 exhibit strong SHG when localized in lipid membranes.^25^ Here we compare the voltage sensitivity of the SHG from JR1 with that from three widely studied styryl dyes (FM4-64, di-4-ANEPPS, and RH237) in hemispherical lipid bilayers (HLBs).^15^, ^26^, ^27^ The results show that the SHG signal from the porphyrin-based dye is exceptionally sensitive to an electric field.

**Figure:**
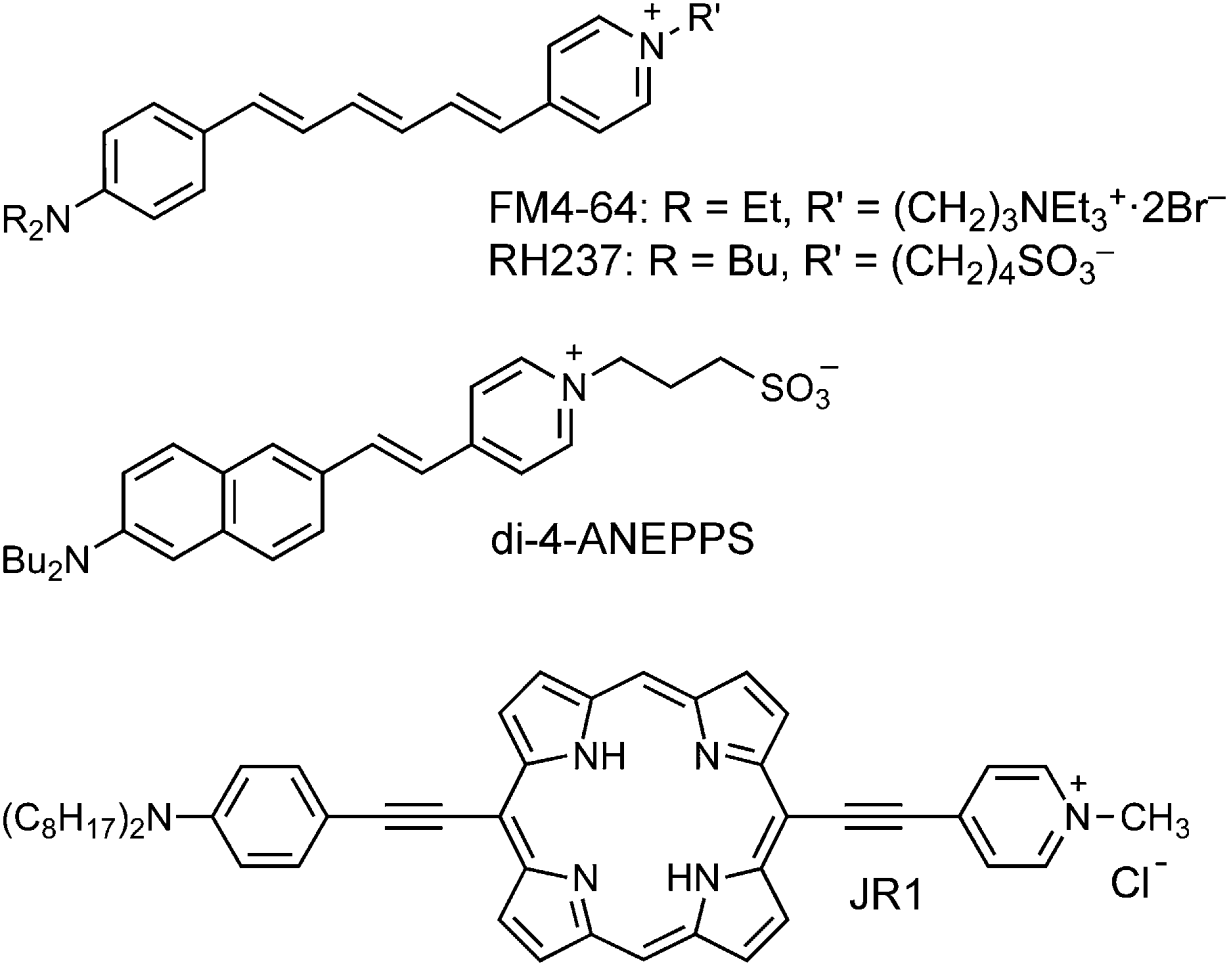


The sensitivity of the porphyrin dye JR1 and the styryl dyes FM4-64, di-4-ANEPPS, and RH237 to transmembrane potential was investigated using the setup shown in Figure 1.^15^, ^26^, ^27^ Each dye was added to an aqueous solution of phosphate buffered saline to give a dye concentration of 5 μm. A solution of diphytanoylphosphatidylcholine (DPhPC, 30 mm) and oxidized cholesterol^28^ (5 mm) in dodecane was then used to create an HLB on the end of the micropipette electrode, containing dye-free buffer. Changes in electrical potential can cause slight movement of the HLB,^29^ so the focal point of the laser (850 nm, 100 fs pulses, 80 MHz) was scanned along the line AB for each applied voltage (*V*_m_). The resulting SHG intensity line scans were stacked to create plots such as those shown for JR1 (Figure 2 a) and FM4-64 (Figure 2 c). It is immediately apparent from these raw data that the modulation in the SHG intensity is stronger with the porphyrin dye. The intensity of the SHG signal (*S*_SHG_) is also plotted in Figure 2 as a function of time, on the same axis as the applied voltage. The shape of the voltage wave-train is more accurately reproduced by the signal generated by JR1 (Figure 2 b) than by FM4-64 (Figure 2 d), and this is confirmed by the normalized cross-correlations of the command-voltage waveforms with the SHG traces (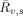 Table 1, see also Figure S2 in the Supporting Information). The responses of all four dyes to a square wave of ±100 mV are compared in Figure 3. The time resolution of these experiments is limited by two factors: the capacitive charging time of the HLB (ca. 2 ms) and the period to complete a return line scan through the bilayer (2–5 ms). The response of JR1 to the applied potential is faster than the time resolution, and appears to be purely electro-optic (expected timescale: sub-picosecond). The responses of RH237, FM4-64, and di-4-ANEPPS are all significantly weaker, with more complex time evolutions. The fast and slow changes in SHG intensity were quantified by averaging the signal at about 13 ms after the voltage step (Δ*S*_fast_/*S*) and at about 350 ms after the voltage step (Δ*S*_slow_/*S*), to give the data in Table 1 and Figure 4.

**Figure 1:**
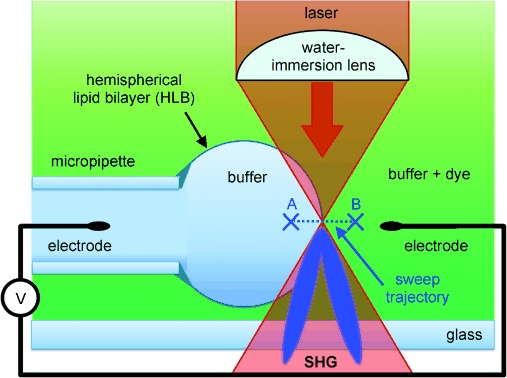
Experimental setup for measuring voltage-dependent SHG (not to scale). The dye binds to the external leaflet of the HLB (diameter ca. 300 μm) and the focal point is scanned repeatedly along the line AB while monitoring the intensity of the SHG signal and applying a range of voltage steps.

**Figure 2:**
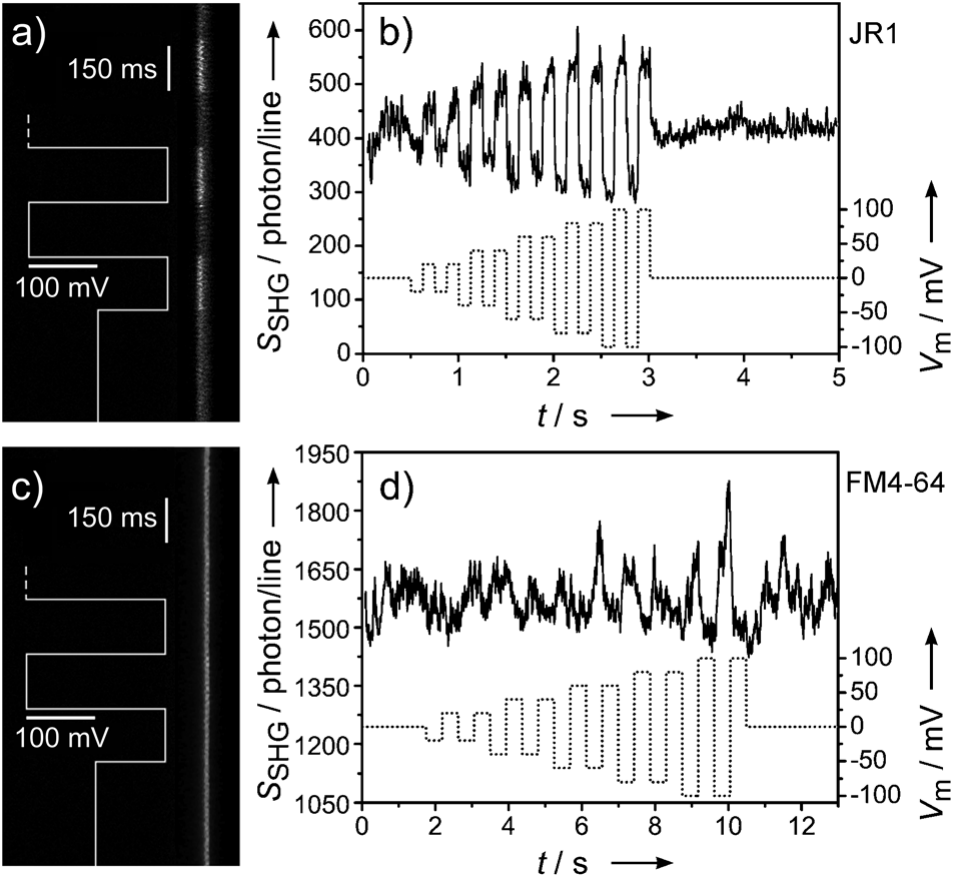
Voltage-dependent SHG traces: a) and b) for porphyrin dye JR1; c) and d) for styryl dye FM4-64. Traces (a) and (c) are stacked line scans (time increasing downwards) while (b) and (d) show how the SHG signal reproduces the applied voltage waveform (dotted curve). Both plots are for representative single traces, without averaging. Traces for di-4-ANEPPS and RH237 may be found in Figure S1 in the Supporting Information.

**Figure 3:**
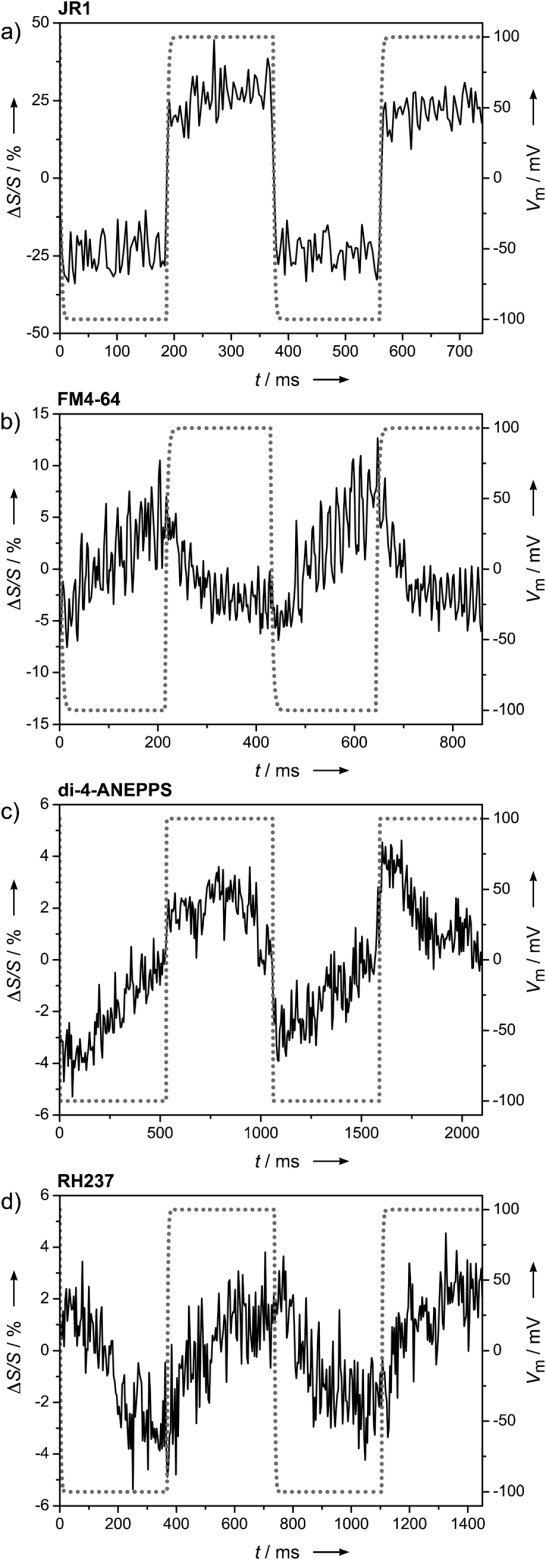
Kinetic traces. Change in SHG intensity caused by an alternating transmembrane potential of *V*_m_=±100 mV (dotted curve) for a) JR1, b) FM4-64, c) di-4-ANEPPS, and d) RH237. Traces (b)–(d) are averages over 4, 3, and 4 experiments, respectively.

**Figure 4:**
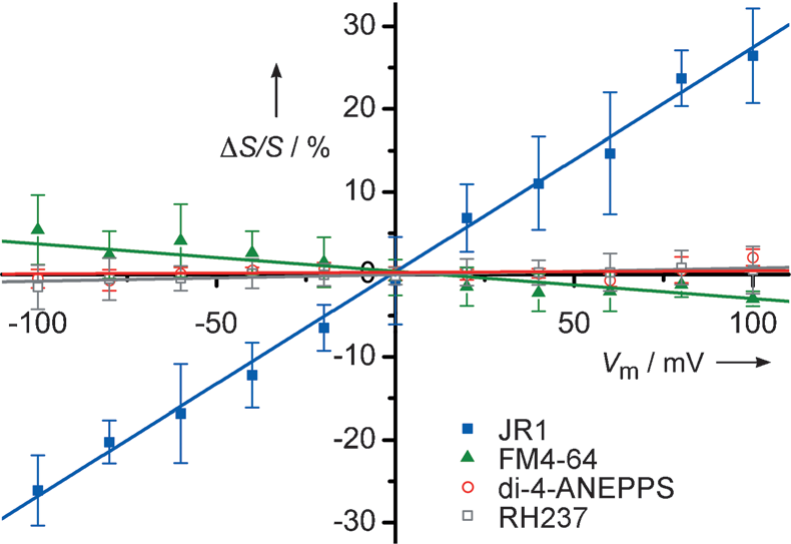
Plot of the change in SHG intensity (Δ*S*_slow_/*S*) against applied potential for JR1 (blue squares), FM4-64 (green triangles), di-4-ANEPPS (red circles) and RH237 (gray squares). The gradients of these lines were used to determine the values of *ΔS*_slow_/*S* (% per 100 mV) in Table 1. (Error bars defined as in Table 1.)

**Table 1:** Performance of voltage-sensitive SHG dyes.

Dye	*ΔS*_fast_/*S*^[a]^ (%/100 mV)	*ΔS*_slow_/*S*^[b]^ (%/100 mV)^[b]^	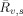 ^[c]^
JR1	+23±4	+27±1	+0.94
FM4-64	+4.1±0.6	−3.0±0.5	−0.69
di-4-ANEPPS	+2.7±0.1	+0.1±0.2	−0.73
RH237	−1.1±0.1	+0.8±0.1	+0.66

[a] Δ*S*_fast_/*S* is the response measured after 8–18 ms. [b] Δ*S*_slow_/*S* is the response measured after 340–360 ms for all dyes except JR1, where it was measured at 90–110 ms. Errors in Δ*S*_fast_/*S* and Δ*S*_slow_/*S* indicate standard errors from at least 6 replicates. [c] 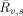 is the normalized cross-correlation, which measures how well the dye reports the applied potential waveform (see Figure S2 in the Supporting Information).

We chose to compare JR1 directly with three previously studied styryl dyes, under identical conditions, because earlier work has shown that a dye’s response to an electric field can depend on experimental parameters such as the composition of the lipid bilayer, the laser wavelength, and the time scale of the measurement.^3^, ^6^, ^19^, ^30^ Our results for FM4-64, di-4-ANEPPS, and RH237 are broadly in line with previously published studies on these dyes, although this is the first time that all three dyes have been compared under identical conditions in HLBs. FM4-64 has been reported to give SHG responses in the range Δ*S*/*S* per 100 mV=5–15 %,^17^–^19^, ^31^–^33^ which is slightly larger than our value (ca. 4 %). Previous studies indicate that the response from FM4-64 contains a fast electro-optic component and a slower component attributed to molecular realignment.^18^, ^32^ The reported SHG response of di-4-ANEPPS at 850 nm is Δ*S*/*S* per 100 mV=18.5 %, with a response time of 71 ms.^20^, ^21^ We detected a small instantaneous electro-optic response for di-4-ANEPPS (Δ*S*/*S* per 100 mV=2.7 %), followed by a slow response with the opposite sign, thereby leading to essentially no overall change in the signal for a time window of 350 ms. This type of cancellation between fast and slow components has been reported previously in other styryl dyes.^34^ RH237 has been reported to give SHG responses in the range Δ*S*/*S* per 100 mV=9–17 %^30^ on a slow time scale (ca. 10 s); under the conditions of our experiment, it is the least responsive of the styryl dyes.

Previous studies have demonstrated that FM4-64 is an effective dye for detecting transmembrane voltages by SHG, and it has already been used to probe action potentials in neurons.^31^–^33^, ^35^, ^36^ Thus the observation that JR1 out-performs FM4-64, giving a fast response which is more than 5 times as voltage sensitive, implies that porphyrin-based dyes could be useful for detecting electrical signals in excitable cells.

The high sensitivity of JR1 to an electric field is probably a consequence of its molecular hyperpolarizability. The intensity of the SHG signal *S* from an ensemble of oriented dye molecules depends quadratically on the first hyperpolarizability of the dye *β*, the number density *N*, and the incident light intensity *I*_0_, according to Equation (1) (where the constant of proportionality *G* accounts for factors such as dye orientation).


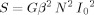
1

In the presence of an electric field *E*, the effective hyperpolarizability *β_E_* is related to the second hyperpolarizability of the dye *γ* by Equation (2).


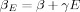
2

Combining Equations (1) and (2) shows that the field sensitivity of the dye is related to its nonlinear optical susceptibilities by Equation (3):


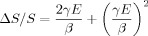
3

We carried out sum-over-state calculations,^37^ in combination with Thomas–Kuhn sum rules analysis^38^, ^39^ to estimate the relevant values of *β*(−2*ω*,*ω*,*ω*) and *γ*(−2*ω*,*ω*,*ω*,0), where *ω* is the photon frequency for JR1 and di-4-ANEPPS at the wavelength of our experiments (850 nm; see the Supporting Information). These two molecules have similar *β* values at 850 nm, whereas *γ* is substantially larger in the porphyrin dye, thus giving calculated field sensitivities of Δ*S*/*S* of 5.5 % for JR1 and 0.72 % for di-4-ANEPPS, for a potential of 100 mV (assuming that the membrane can be approximated to an insulating slab of thickness 3 nm^40^). Although these calculations involve a number of approximations, they reproduce the observed trend in field sensitivity, thus indicating that the fast signal from JR1 (*ΔS*/*S* of 23 % per 100 mV) is an electro-optic response originating from its large second hyperpolarizability. The greater voltage sensitivity of JR1 compared with the styryl dyes can be seen as a manifestation of the scaling behaviors of nonlinear optical susceptibilities; there is no simple scaling law, but the ratio *γ*/*β* generally increases with the size of the π system.^41^–^44^ Equation (3) indicates that, in general, the sensitivity of a dye to electric field (Δ*S*/*S*) will increase as the π systems becomes larger and more polarizable.

The high polarizability of porphyrin-based π systems, and the scope for strong resonance enhancements from transitions with high oscillator strengths, gives rise to a rich variety of nonlinear optical effects including large first and second hyperpolarizabilities.^45^–^51^ Conjugated porphyrin dimers exhibit much larger third-order susceptibilities (*γ*) than the corresponding porphyrin monomers,^49^–^51^ thus Equation (3) implies that dimeric analogues of JR1 will display even higher sensitivities to electric field. Previous studies on voltage-sensitive SHG dyes have focused entirely on styryl and retinal chromophores. The results reported here show the JR1 out-performs these dyes by a factor of 5–10, and indicate that porphyrin-based dyes may be useful for probing fast changes in membrane potential.
